# 4-{2-[(*Z*)-(5-Methyl-2-fur­yl)methyl­idene­amino]­eth­yl}benzene­sulfonamide

**DOI:** 10.1107/S1600536810034045

**Published:** 2010-08-28

**Authors:** Khalid Mahmood, Muhammad Yaqub, M. Nawaz Tahir, Zahid Shafiq, Ashfaq Mahmood Qureshi

**Affiliations:** a Department of Chemistry, Bahauddin Zakariya University, Multan 60800, Pakistan; b Department of Physics, University of Sargodha, Sargodha, Pakistan

## Abstract

In the title compound, C_14_H_16_N_2_O_3_S, the dihedral angle between the phenyl and 5-methyl­furan groups is 54.89 (14)° and the C=N bond assumes a *trans* conformation. In the crystal, inversion dimers linked by pairs of N—H⋯O hydrogen bonds generate *R*_2_^2^(8) ring motifs. The dimers are inter­linked by N—H⋯N hydrogen bonds, resulting in the formation of infinite chains extending along the *b* axis. The packing is consolidated by weak C—H⋯π inter­actions.

## Related literature

For biochemical background and related crystal structures, see: Chohan *et al.* (2008 ▶); Davis *et al.* (2007 ▶); Li (2006 ▶); Suo (2008 ▶); For graph-set notation, see: Bernstein *et al.* (1995 ▶).
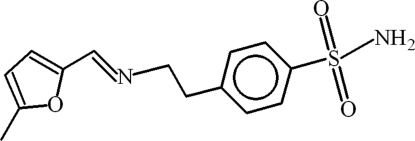


## Experimental

### 

#### Crystal data

C_14_H_16_N_2_O_3_S
*M* *_r_* = 292.35
Triclinic, 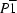

*a* = 9.1947 (14) Å
*b* = 9.7592 (14) Å
*c* = 9.8493 (15) Å
α = 61.027 (6)°
β = 70.650 (6)°
γ = 81.574 (7)°
*V* = 729.4 (2) Å^3^
*Z* = 2
Mo *K*α radiation
μ = 0.23 mm^−1^
*T* = 296 K
0.20 × 0.16 × 0.08 mm

#### Data collection

Bruker Kappa APEXII CCD diffractometer
Absorption correction: multi-scan (*SADABS*; Bruker, 2005 ▶) *T*_min_ = 0.956, *T*_max_ = 0.977
10058 measured reflections
2638 independent reflections
1461 reflections with *I* > 2σ(*I*)
*R*_int_ = 0.068

#### Refinement

*R*[*F* ^2^ > 2σ(*F* ^2^)] = 0.058
*wR*(*F* ^2^) = 0.153
*S* = 0.97
2638 reflections
188 parameters
H atoms treated by a mixture of independent and constrained refinement
Δρ_max_ = 0.22 e Å^−3^
Δρ_min_ = −0.33 e Å^−3^

### 

Data collection: *APEX2* (Bruker, 2009 ▶); cell refinement: *SAINT* (Bruker, 2009 ▶); data reduction: *SAINT*; program(s) used to solve structure: *SHELXS97* (Sheldrick, 2008 ▶); program(s) used to refine structure: *SHELXL97* (Sheldrick, 2008 ▶); molecular graphics: *ORTEP-3 for Windows* (Farrugia, 1997 ▶) and *PLATON* (Spek, 2009 ▶); software used to prepare material for publication: *WinGX* (Farrugia, 1999 ▶) and *PLATON*.

## Supplementary Material

Crystal structure: contains datablocks global, I. DOI: 10.1107/S1600536810034045/hb5617sup1.cif

Structure factors: contains datablocks I. DOI: 10.1107/S1600536810034045/hb5617Isup2.hkl

Additional supplementary materials:  crystallographic information; 3D view; checkCIF report

## Figures and Tables

**Table 1:** Hydrogen-bond geometry (Å, °) *Cg*1 and *Cg*2 are the centroids of furan (C10—C13/O3) and phenyl (C1—C6) rings, respectively.

*D*—H⋯*A*	*D*—H	H⋯*A*	*D*⋯*A*	*D*—H⋯*A*
N1—H1*A*⋯O1^i^	0.80 (4)	2.18 (4)	2.928 (5)	155 (4)
N1—H1*B*⋯N2^ii^	0.82 (4)	2.25 (5)	3.015 (5)	156 (5)
C6—H6⋯*Cg*1^iii^	0.93	2.87	3.596 (4)	136
C11—H11⋯*Cg*2^iv^	0.93	2.75	3.535 (4)	143
C14—H14*C*⋯*Cg*2^v^	0.96	2.84	3.743 (5)	157

Symmetry codes: (i) 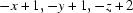; (ii) 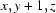; (iii) 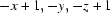; (iv) 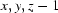; (v) 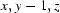.

**Fig. 1.**
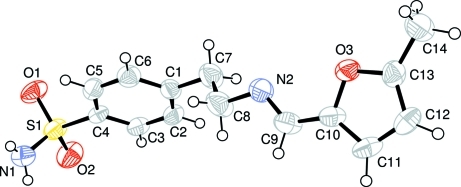
View of (I) with displacement ellipsoids drawn at the 50% probability level.

**Fig. 2.**
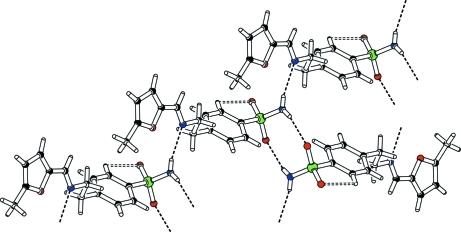
The partial packing of (I), which shows that molecules form dimers which are interlinked.

## supplementary crystallographic information

### Comment

The title compound (I, Fig. 1) is being reported here in the context of our new project of synthesizing Schiff basis of various sulfonamide drugs, studying their bio-activity and the formation of their metal complexes.

The crystal structures of (II) 4-(2-(3-ethyl-4-methyl-2-oxo-3-pyrrolidine-1-carboxamido)ethyl) benzenesulfonamide (Li, 2006), (III) *N*-(2-(4-(aminosulfonyl)phenyl)ethyl)-2-(4-hydroxyphenyl)acetamide (Davis *et al.*, 2007), (IV) 4-(2-((5-chloro-2-hydroxybenzylidene)amino)ethyl)benzenesulfonamide (Chohan *et al.*, 2008) and (V) (*E*)-4-[(5-methyl-2-furyl)methyleneamino]benzenesulfonic acid (Suo, 2008) have been published which are related to the title compound.

In (I), the thiophenol A (C1–C6/S1) and 5-methylfuran-2-yl B (C10–C14/O3) are planar with r. m. s. deviations of 0.0037 and 0.0029 Å, respectively. The dihedral angle between A/B is 54.89 (14)°. The S-atom is at a distance of -0.4487 (19) Å from the plane formed by (O1/O2/N1). The central group of *N*-methylideneethanamine makes a torsion angle of -136.6 (4)°. In the title compound an S(5) ring motif (Bernstein *et al.*, 1995) is formed due to C—H···O type of intramolecular H-bonding. The molecules are dimerized due to N—H···O type of intermolecular H-bonding (Table 1, Fig. 2) with *R*_2_^2^(8) ring motif. The dimers are interlinked through H-bondings of N—H···N type resulting in the formation of infinite one dimensional polymeric chains extending along the *b* axis. In the stabilization of molecules C—H···π interactions (Table 1) play an important role.

### Experimental

An ethanol solution (15 ml) of sulfonamide (0.20 g, 1 mmol) was added to the solution of 5-methylfuran-2-carbaldehyde (0.099 ml, 1 mmol) in ethanol (10 ml). The reaction mixture was refluxed for 4 h. The solution was cooled to room temperature, filtered and volume reduced to about one-third using rotary evaporator. It was then allowed to stand for 5 days, after which dark yellow plates of (I) were obtained.

### Refinement

The coordinates of H-atoms of amine were refined and the other H-atoms were positioned geometrically (C–H = 0.93–0.97 Å) and refined as riding with *U*_iso_(H) = *xU*_eq_(C, N), where *x* = 1.5 for methyl and *x* = 1.2 for all other H-atoms.

### Crystal data

**Table:**

C_14_H_16_N_2_O_3_S	*Z* = 2
*M**_r_* = 292.35	*F*(000) = 308
Triclinic, *P*1	*D*_x_ = 1.331 Mg m^−^^3^
Hall symbol: -P 1	Mo *K*α radiation, λ = 0.71073 Å
*a* = 9.1947 (14) Å	Cell parameters from 1461 reflections
*b* = 9.7592 (14) Å	θ = 2.4–25.3°
*c* = 9.8493 (15) Å	µ = 0.23 mm^−^^1^
α = 61.027 (6)°	*T* = 296 K
β = 70.650 (6)°	Plate, dark yellow
γ = 81.574 (7)°	0.20 × 0.16 × 0.08 mm
*V* = 729.4 (2) Å^3^	

### Data collection

**Table:**

Bruker Kappa APEXII CCD diffractometer	2638 independent reflections
Radiation source: fine-focus sealed tube	1461 reflections with *I* > 2σ(*I*)
graphite	*R*_int_ = 0.068
Detector resolution: 8.20 pixels mm^-1^	θ_max_ = 25.5°, θ_min_ = 2.4°
ω scans	*h* = −11→11
Absorption correction: multi-scan (*SADABS*; Bruker, 2005)	*k* = −11→11
*T*_min_ = 0.956, *T*_max_ = 0.977	*l* = −11→11
10058 measured reflections	

### Refinement

**Table:**

Refinement on *F*^2^	Primary atom site location: structure-invariant direct methods
Least-squares matrix: full	Secondary atom site location: difference Fourier map
*R*[*F*^2^ > 2σ(*F*^2^)] = 0.058	Hydrogen site location: inferred from neighbouring sites
*wR*(*F*^2^) = 0.153	H atoms treated by a mixture of independent and constrained refinement
*S* = 0.97	*w* = 1/[σ^2^(*F*_o_^2^) + (0.0756*P*)^2^] where *P* = (*F*_o_^2^ + 2*F*_c_^2^)/3
2638 reflections	(Δ/σ)_max_ < 0.001
188 parameters	Δρ_max_ = 0.22 e Å^−^^3^
0 restraints	Δρ_min_ = −0.33 e Å^−^^3^

### Special details

**Table:**

Geometry. Bond distances, angles *etc*. have been calculated using the rounded fractional coordinates. All su's are estimated from the variances of the (full) variance-covariance matrix. The cell e.s.d.'s are taken into account in the estimation of distances, angles and torsion angles
Refinement. Refinement of *F*^2^ against ALL reflections. The weighted *R*-factor *wR* and goodness of fit *S* are based on *F*^2^, conventional *R*-factors *R* are based on *F*, with *F* set to zero for negative *F*^2^. The threshold expression of *F*^2^ > σ(*F*^2^) is used only for calculating *R*-factors(gt) *etc*. and is not relevant to the choice of reflections for refinement. *R*-factors based on *F*^2^ are statistically about twice as large as those based on *F*, and *R*- factors based on ALL data will be even larger.

### Fractional atomic coordinates and isotropic or equivalent isotropic displacement parameters (Å^2^)

**Table:**

	*x*	*y*	*z*	*U*_iso_*/*U*_eq_	
S1	0.74690 (11)	0.53303 (11)	0.83986 (11)	0.0552 (4)	
O1	0.6857 (3)	0.4598 (3)	1.0147 (3)	0.0670 (10)	
O2	0.8947 (3)	0.6089 (3)	0.7640 (3)	0.0748 (11)	
O3	0.7176 (3)	−0.2510 (3)	0.3423 (3)	0.0507 (8)	
N1	0.6254 (4)	0.6587 (4)	0.7715 (4)	0.0646 (12)	
N2	0.7059 (3)	−0.0369 (3)	0.4601 (4)	0.0574 (11)	
C1	0.7622 (4)	0.1521 (4)	0.6961 (4)	0.0461 (11)	
C2	0.8939 (4)	0.2404 (4)	0.6471 (4)	0.0510 (12)	
C3	0.8887 (4)	0.3549 (4)	0.6906 (4)	0.0474 (11)	
C4	0.7533 (4)	0.3868 (4)	0.7848 (4)	0.0413 (11)	
C5	0.6216 (4)	0.2987 (4)	0.8352 (4)	0.0486 (12)	
C6	0.6275 (4)	0.1842 (4)	0.7922 (4)	0.0510 (12)	
C7	0.7667 (4)	0.0281 (4)	0.6470 (5)	0.0635 (14)	
C8	0.7022 (5)	0.0834 (4)	0.5068 (5)	0.0725 (18)	
C9	0.7504 (4)	0.0059 (4)	0.3090 (5)	0.0568 (14)	
C10	0.7612 (4)	−0.0949 (4)	0.2409 (4)	0.0496 (12)	
C11	0.8112 (4)	−0.0715 (5)	0.0851 (4)	0.0594 (16)	
C12	0.8012 (4)	−0.2149 (5)	0.0879 (4)	0.0615 (16)	
C13	0.7442 (4)	−0.3207 (4)	0.2452 (4)	0.0504 (12)	
C14	0.7067 (5)	−0.4902 (4)	0.3273 (5)	0.0703 (16)	
H1A	0.535 (4)	0.638 (5)	0.802 (5)	0.0777*	
H1B	0.643 (4)	0.724 (5)	0.676 (5)	0.0777*	
H2	0.98644	0.22087	0.58379	0.0612*	
H3	0.97774	0.41220	0.65631	0.0567*	
H5	0.52913	0.31836	0.89844	0.0585*	
H6	0.53869	0.12590	0.82844	0.0611*	
H7A	0.70791	−0.06283	0.73939	0.0768*	
H7B	0.87257	−0.00344	0.61620	0.0768*	
H8A	0.59655	0.11552	0.53728	0.0866*	
H8B	0.76142	0.17392	0.41410	0.0866*	
H9	0.77791	0.11076	0.23804	0.0682*	
H11	0.84604	0.02315	−0.00750	0.0717*	
H12	0.82872	−0.23341	−0.00147	0.0735*	
H14A	0.74691	−0.53007	0.25033	0.1053*	
H14B	0.59683	−0.50553	0.37049	0.1053*	
H14C	0.75202	−0.54468	0.41435	0.1053*	

### Atomic displacement parameters (Å^2^)

**Table:**

	*U*^11^	*U*^22^	*U*^33^	*U*^12^	*U*^13^	*U*^23^
S1	0.0661 (7)	0.0559 (6)	0.0413 (6)	−0.0178 (5)	−0.0026 (4)	−0.0246 (5)
O1	0.0882 (19)	0.0783 (18)	0.0346 (14)	−0.0201 (14)	−0.0061 (13)	−0.0285 (13)
O2	0.0692 (17)	0.0801 (19)	0.0763 (19)	−0.0301 (14)	−0.0002 (14)	−0.0428 (16)
O3	0.0698 (16)	0.0453 (14)	0.0336 (13)	−0.0044 (11)	−0.0145 (11)	−0.0152 (11)
N1	0.073 (2)	0.050 (2)	0.052 (2)	−0.0051 (18)	−0.004 (2)	−0.0174 (16)
N2	0.081 (2)	0.0476 (18)	0.046 (2)	0.0008 (15)	−0.0207 (16)	−0.0225 (16)
C1	0.064 (2)	0.0387 (19)	0.038 (2)	0.0061 (17)	−0.0210 (18)	−0.0175 (16)
C2	0.058 (2)	0.053 (2)	0.034 (2)	0.0076 (18)	−0.0114 (17)	−0.0176 (18)
C3	0.053 (2)	0.047 (2)	0.0345 (19)	−0.0092 (16)	−0.0091 (16)	−0.0132 (17)
C4	0.044 (2)	0.0443 (19)	0.0272 (17)	−0.0032 (15)	−0.0060 (15)	−0.0124 (15)
C5	0.048 (2)	0.050 (2)	0.046 (2)	−0.0018 (16)	−0.0060 (16)	−0.0255 (18)
C6	0.052 (2)	0.049 (2)	0.050 (2)	−0.0070 (16)	−0.0112 (18)	−0.0221 (19)
C7	0.089 (3)	0.052 (2)	0.060 (2)	0.009 (2)	−0.032 (2)	−0.030 (2)
C8	0.118 (4)	0.049 (2)	0.064 (3)	0.008 (2)	−0.042 (2)	−0.029 (2)
C9	0.073 (3)	0.042 (2)	0.052 (2)	−0.0064 (18)	−0.025 (2)	−0.0135 (19)
C10	0.060 (2)	0.043 (2)	0.037 (2)	−0.0075 (16)	−0.0156 (16)	−0.0092 (17)
C11	0.069 (3)	0.062 (3)	0.031 (2)	−0.0100 (19)	−0.0118 (17)	−0.0085 (18)
C12	0.070 (3)	0.078 (3)	0.038 (2)	0.000 (2)	−0.0145 (18)	−0.029 (2)
C13	0.061 (2)	0.055 (2)	0.046 (2)	0.0056 (17)	−0.0223 (18)	−0.029 (2)
C14	0.105 (3)	0.054 (2)	0.062 (3)	0.004 (2)	−0.040 (2)	−0.026 (2)

### Geometric parameters (Å, °)

**Table:**

S1—O1	1.441 (3)	C10—C11	1.357 (5)
S1—O2	1.428 (3)	C11—C12	1.402 (7)
S1—N1	1.582 (4)	C12—C13	1.346 (5)
S1—C4	1.743 (4)	C13—C14	1.482 (6)
O3—C10	1.385 (5)	C2—H2	0.9300
O3—C13	1.365 (5)	C3—H3	0.9300
N2—C8	1.446 (6)	C5—H5	0.9300
N2—C9	1.265 (5)	C6—H6	0.9300
N1—H1B	0.82 (4)	C7—H7A	0.9700
N1—H1A	0.80 (4)	C7—H7B	0.9700
C1—C6	1.387 (5)	C8—H8A	0.9700
C1—C7	1.496 (6)	C8—H8B	0.9700
C1—C2	1.399 (6)	C9—H9	0.9300
C2—C3	1.367 (6)	C11—H11	0.9300
C3—C4	1.382 (5)	C12—H12	0.9300
C4—C5	1.395 (6)	C14—H14A	0.9600
C5—C6	1.363 (6)	C14—H14B	0.9600
C7—C8	1.509 (6)	C14—H14C	0.9600
C9—C10	1.412 (6)		
			
O1—S1—O2	118.85 (17)	C12—C13—C14	132.9 (4)
O1—S1—N1	106.50 (18)	C1—C2—H2	119.00
O1—S1—C4	106.27 (18)	C3—C2—H2	120.00
O2—S1—N1	107.94 (19)	C2—C3—H3	119.00
O2—S1—C4	108.41 (19)	C4—C3—H3	119.00
N1—S1—C4	108.5 (2)	C4—C5—H5	120.00
C10—O3—C13	107.0 (3)	C6—C5—H5	120.00
C8—N2—C9	116.4 (4)	C1—C6—H6	119.00
S1—N1—H1B	123 (3)	C5—C6—H6	119.00
H1A—N1—H1B	105 (4)	C1—C7—H7A	109.00
S1—N1—H1A	122 (3)	C1—C7—H7B	109.00
C6—C1—C7	121.5 (4)	C8—C7—H7A	109.00
C2—C1—C6	117.2 (4)	C8—C7—H7B	109.00
C2—C1—C7	121.3 (3)	H7A—C7—H7B	108.00
C1—C2—C3	121.1 (4)	N2—C8—H8A	109.00
C2—C3—C4	121.1 (4)	N2—C8—H8B	109.00
S1—C4—C3	121.0 (3)	C7—C8—H8A	109.00
C3—C4—C5	118.3 (4)	C7—C8—H8B	109.00
S1—C4—C5	120.7 (3)	H8A—C8—H8B	108.00
C4—C5—C6	120.4 (4)	N2—C9—H9	118.00
C1—C6—C5	121.9 (4)	C10—C9—H9	118.00
C1—C7—C8	112.3 (4)	C10—C11—H11	126.00
N2—C8—C7	112.2 (4)	C12—C11—H11	126.00
N2—C9—C10	124.4 (4)	C11—C12—H12	127.00
O3—C10—C9	119.6 (3)	C13—C12—H12	127.00
O3—C10—C11	108.0 (4)	C13—C14—H14A	109.00
C9—C10—C11	132.4 (4)	C13—C14—H14B	109.00
C10—C11—C12	108.2 (3)	C13—C14—H14C	109.00
C11—C12—C13	106.7 (4)	H14A—C14—H14B	110.00
O3—C13—C12	110.1 (4)	H14A—C14—H14C	110.00
O3—C13—C14	117.0 (3)	H14B—C14—H14C	109.00
			
O1—S1—C4—C3	−125.4 (3)	C2—C1—C7—C8	−100.7 (4)
O1—S1—C4—C5	54.5 (3)	C6—C1—C7—C8	79.4 (5)
O2—S1—C4—C3	3.4 (4)	C1—C2—C3—C4	0.2 (5)
O2—S1—C4—C5	−176.7 (3)	C2—C3—C4—S1	−179.9 (3)
N1—S1—C4—C3	120.4 (3)	C2—C3—C4—C5	0.2 (5)
N1—S1—C4—C5	−59.7 (3)	S1—C4—C5—C6	−179.8 (3)
C13—O3—C10—C9	−178.2 (4)	C3—C4—C5—C6	0.1 (5)
C13—O3—C10—C11	0.8 (4)	C4—C5—C6—C1	−0.9 (6)
C10—O3—C13—C12	−0.5 (4)	C1—C7—C8—N2	−179.7 (3)
C10—O3—C13—C14	179.7 (4)	N2—C9—C10—O3	1.7 (6)
C9—N2—C8—C7	−136.6 (4)	N2—C9—C10—C11	−177.1 (4)
C8—N2—C9—C10	−179.9 (4)	O3—C10—C11—C12	−0.8 (5)
C6—C1—C2—C3	−0.9 (5)	C9—C10—C11—C12	178.0 (4)
C7—C1—C2—C3	179.2 (3)	C10—C11—C12—C13	0.5 (5)
C2—C1—C6—C5	1.3 (5)	C11—C12—C13—O3	0.0 (5)
C7—C1—C6—C5	−178.8 (3)	C11—C12—C13—C14	179.8 (4)

### Hydrogen-bond geometry (Å, °)

**Table:**

Cg1 and Cg2 are the centroids of furan (C10—C13/O3) and phenyl (C1—C6) rings, respectively.

**Table:**

*D*—H···*A*	*D*—H	H···*A*	*D*···*A*	*D*—H···*A*
N1—H1A···O1^i^	0.80 (4)	2.18 (4)	2.928 (5)	155 (4)
N1—H1B···N2^ii^	0.82 (4)	2.25 (5)	3.015 (5)	156 (5)
C6—H6···Cg1^iii^	0.93	2.87	3.596 (4)	136
C11—H11···Cg2^iv^	0.93	2.75	3.535 (4)	143
C14—H14C···Cg2^v^	0.96	2.84	3.743 (5)	157

Symmetry codes: (i) −*x*+1, −*y*+1, −*z*+2; (ii) *x*, *y*+1, *z*; (iii) −*x*+1, −*y*, −*z*+1; (iv) *x*, *y*, *z*−1; (v) *x*, *y*−1, *z*.
